# Effect of thyroid stimulating hormone on the prognosis of coronary heart disease

**DOI:** 10.3389/fendo.2025.1433106

**Published:** 2025-02-17

**Authors:** Ning Ding, Rui Hua, Hanqing Guo, Yu Xu, Zuyi Yuan, Yue Wu, Ting Li

**Affiliations:** ^1^ Department of Cardiovascular Medicine, The First Affiliated Hospital, Xi’an Jiaotong University, Xi’an, Shaanxi, China; ^2^ Key Laboratory of Molecular Cardiology, Xi'an Jiaotong University, Xi’an, Shaanxi, China; ^3^ Key Laboratory of Environment and Genes Related to Diseases, Ministry of Education, Xi’an, Shaanxi, China; ^4^ Department of Gastroenterology, Xi’an Central Hospital, Xi’an Jiaotong University, Xi’an, Shaanxi, China

**Keywords:** thyroid stimulating hormone, coronary-heart-disease, major adverse cardiovascular events, mortality, prognosis

## Abstract

**Introduction:**

Clinical studies have shown that thyroid stimulating hormone (TSH) is associated with increased cardiovascular disease risk and mortality. Even within normal ranges, elevated TSH levels have an impact on the cardiovascular system and have been associated with cardiac dysfunction. The aim of our study was to evaluate the predictive value of admission fasting serum TSH levels in patients with coronary heart disease in relation to long-term major adverse cardiovascular events (MACE) and all-cause mortality.

**Method:**

A total of 3515 patients with coronary heart disease who met the inclusion criteria were divided into four groups according to the quantile of TSH levels: Group 1 (TSH, 0.34-1.02 mIU/L, n=878); Group 2 (TSH, 1.03-1.71 mIU/L, n=886); Group 3 (TSH, 1.72-2.84 mIU/L, n=880); and Group 4 (TSH, 2.86-5.50 mIU/L, n=873). MACE and all-cause mortality were also compared. TSH concentrations associated with the risk of MACE, all-cause mortality were assessed using continuous scales (restricted quartic splines) and Cox proportional hazards regression models.

**Results:**

A total of 3515 patients with coronary heart disease were eligible for analysis. At a median follow-up of 70 months, patients in group 2 had a lower incidence of MACE compared to the other three groups. All-cause mortality was lower in the 3rd group. Restricted quartic spline analysis also revealed that TSH concentrations were associated with heart failure risk.

**Discussion:**

TSH levels have predictive value for adverse cardiovascular events and heart failure in patients with coronary heart disease.

## Graphical Abstract

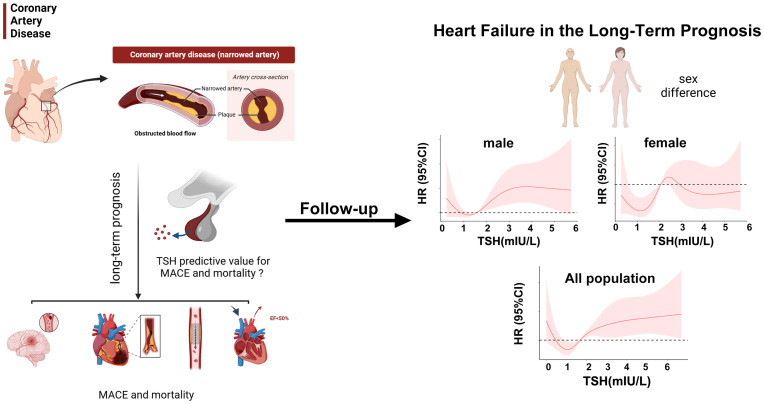

## Introduction

Coronary heart disease (CHD), the main cause of ischemic heart disease, is one of the major cardiovascular diseases threatening the global human health. In 2019, heart disease was the top cause of disability-adjusted life year in the 50-year-and-older age group (1, 2). CHD progression is dynamic and unpredictable and can accidentally lead to major adverse cardiovascular events (MACE), such as myocardial infarction (MI), revascularization, heart failure, stroke and cardiovascular death. It is particularly concerning that patients remain at high risk of MACE despite revascularization and optimal secondary prevention according to the current guidelines (3–6). Thus, additional risk stratification models, including sensitive biomarkers and clinical indicators, are needed to identify high-risk patients for accurate secondary prevention of CHD.

The role of thyroid hormones in triggering and exacerbating potential cardiovascular disease has been increasingly recognized, and the use of thyroid function status as a new risk factor for cardiovascular events has attracted increasing attention (7–10). Previous studies have reported that minor fluctuations in thyroid hormone levels have a detrimental impact on the cardiovascular system (11–13). TSH levels are the most sensitive indicator of thyroid function. TSH levels are correlated with an increased risk of cardiovascular morbidity and mortality (14–18). Recent studies have also indicated that even TSH concentrations within normal range may have influence on cardiovascular outcomes. In particular, persistent hypothyroidism leads to increased endothelial dysfunction and decreased left ventricular function (19). In addition, TSH levels in the upper part of the reference range are related to a worse cardiovascular risk profile, including systolic and diastolic blood pressure, body mass index, coronary or carotid atherosclerosis, and a higher risk of mortality, MACE (HR, 1.06 per additional 1 mIU/L) and heart failure (9, 13, 20, 21). In animal models, cells in the vascular wall are directly influenced by thyroid hormones. A higher triiodothyronine concentration leads to the relaxation of vascular smooth muscle cells, upregulation of vascular resistance, dysfunction of endothelial cells, and increased cardiac contractility (22–24). These findings indicate that thyroid function status is a highly important risk factor for predicting cardiovascular events.

To date, it is unclear whether TSH levels within the reference range have predictive value for long-term prognosis in patients with chronic coronary heart disease. In this study, we aimed to investigate the association between normal TSH levels and the long-term incidence of MACE and all cause mortality in patients diagnosed with CHD.

## Methods

### Study population

From January 2013 to July 2020, 4016 consecutive coronary artery disease patients were admitted to the cardiology department of the First Affiliated Hospital of Xi’an Jiaotong University. Only patients with TSH levels within the reference range (0.34 to 5.50 mIU/L) were eligible for analysis. Patients were divided into four groups according to the tertile of the TSH levels. The exclusion criteria consisted of 1) missing thyroid function test results (n = 59); 2) abnormal thyroid status and TSH above the reference range (n = 397); 3) prior or current thyroid disease (including prior history, surgery, or drug therapy for thyroid disease) (n = 132); and 4) receiving steroids and amiodarone before admission (n = 44).

The detailed demographic, clinical, drug, hematologic, and angiographic data were obtained from the medical records. The demographic variables included respondent age, sex, race/ethnicity, and education status. Smoking status; history of cancer, diabetes, hypertension, or dyslipidemia; and receipt of a statin prescription were self-reported. Weight and height were measured and used to calculate body mass index (BMI; calculated as weight in kilograms divided by height in meters squared).

Patients were treated according to standard clinical guidelines. Our study was conducted in accordance with the Declaration of Helsinki and was approved by the Ethics Committee of the First Affiliated Hospital of Xi’an Jiaotong University. Written informed consent was obtained from all study participants.

CHD was diagnosed on the basis of the presence of at least 50% coronary stenosis in at least one major coronary artery according to the CAG results assessed by at least two experienced interventional cardiologists.

### Thyroid function

**Figure 1:**
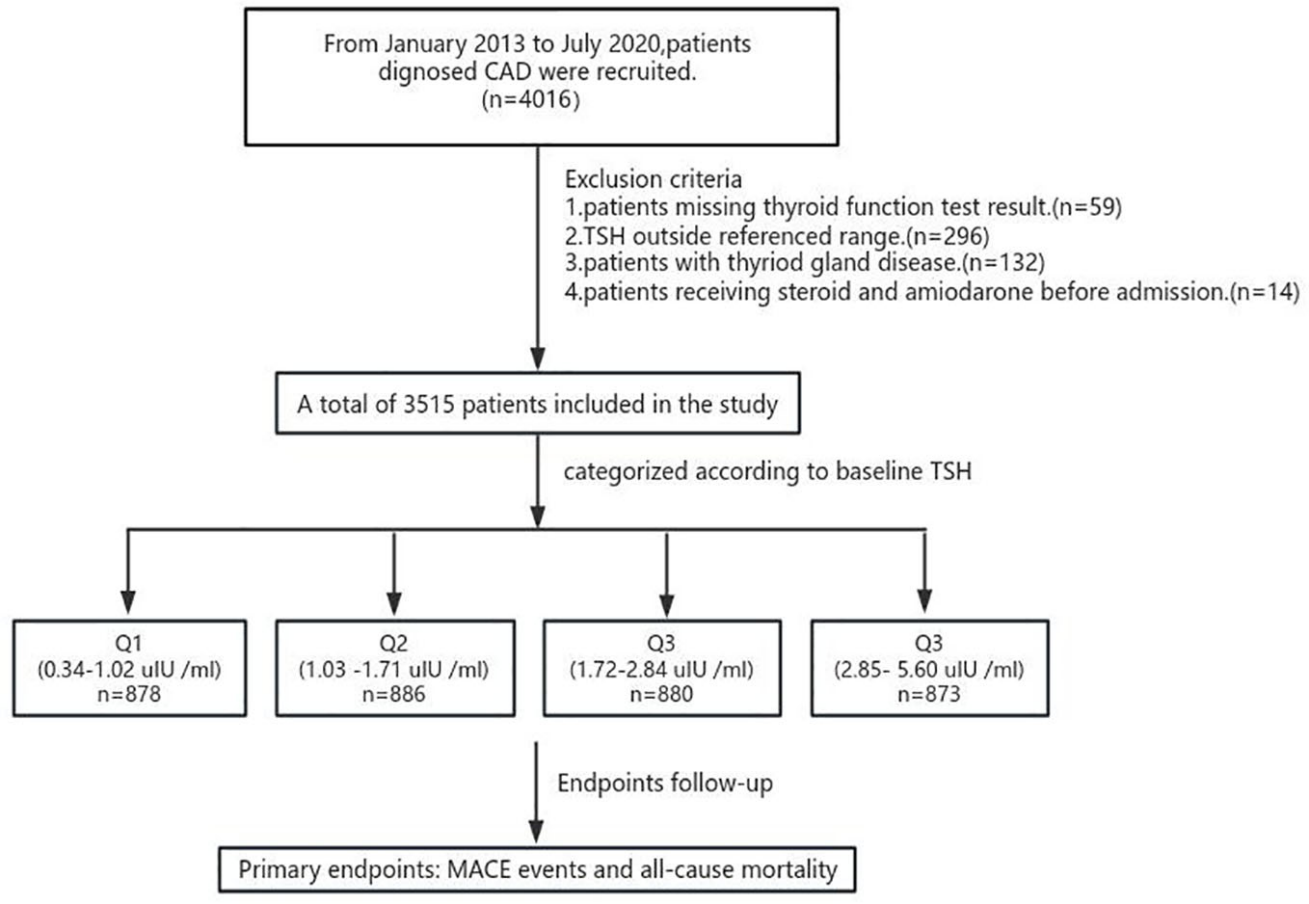
The study flowchart. CHD, coronary heart disease; TSH, thyroid-stimulating hormone(mIU/L=uIU/ml).

Blood samples were collected within 24 h of hospital admission. The thyroid function test included serum TSH, free triiodothyronine (FT3), and free thyroxine (FT4) levels. The normal ranges for TSH and FT4 were defined as 0.34 to 5.50 mIU/L and 0.6 to 1.6 ng/dL, respectively. 3515 Participants with serum TSH and FT4 concentrations within the normal range were considered to be euthyroid. Given the potential U-curve association between TSH and MACE, a total of 3515 patients with coronary heart disease who met the inclusion criteria were divided into four groups according to the quantile of TSH levels: Q1 (TSH, 0.34-1.02 mIU/L, n=878); Q2 (TSH, 1.03-1.71 mIU/L, n=886; Q3 (TSH, 1.72-2.84 mIU/L, n=880; and Q4 (TSH, 2.86-5.50 mIU/L, n=873) (**Figure 1**).

### Outcome ascertainment

The primary endpoint was major cardiovascular adverse events (MACE), including all-cause death, myocardial infarction, revascularization, and heart failure. The secondary endpoints included all-cause death and stroke. A myocardial infarction event was defined as a nonfatal myocardial infarction or cardiac or muscle infarction-related death diagnosed by symptoms and signs; a revascularization event was defined as a secondary hospitalization or death after percutaneous or cutaneous coronary intervention or coronary artery bypass grafting; and a heart failure event was defined as any heart failure-related hospitalization or death. Stroke events were defined as hospitalization or death related to ischemic or nonischemic stroke. The time to event was calculated from the day of TSH measurement to the end of follow-up and the date of death and MACE.

### Statistical analysis

The mean and standard deviation were calculated for continuous variables, and the proportion was calculated for categorical variables in each category according to the TSH concentration. Categorical variables are shown as frequencies and percentages. The Shapiro–Wilk normality test was performed to test the normality of the data. The means of continuous variables were compared using one-way analysis of the Kruskal–Wallis test. Analysis was performed using SPSS 26 statistical analysis software. ANOVA was used for comparisons between multiple groups, and the rank-sum test was used for comparisons of variables with uneven variance; categorical variables are expressed as frequencies (percentages), and the χ2 test or Fisher’s exact test was used. Univariate and multivariate Cox regression models were used to analyze the risk of composite cardiovascular adverse events and all-cause death in patients with CHD with different thyroid function. The Kaplan-Meier method was used to construct patient survival curves and lines, and comparisons between groups were performed with the log-rank test. The associations between TSH concentration in the reference range and MACE events and all-cause mortality were evaluated on a continuous scale with restricted cubic spline curves based on Cox proportional hazards models with 4 nodes at the 5th, 35th, 65th and 95th percentiles of TSH (25); restricted cubic spline curves were rerestricted by sex stratification with 4 nodes at the 5th, 35th, 65th and 95th percentiles of TSH by the R package.

## Results

### Baseline data

A total of 3515 consecutive patients were enrolled and divided into four groups according to the quartile of TSH levels: Q1 (TSH, 0.34-1.02 mIU/L, n=878), Q2 (TSH, 1.03-1.71 mIU/L, n=886), Q3 (TSH, 1.72-2.84 mIU/L, n=880) and Q4 (TSH, 2.86-5.50 mIU/L, n=873)]. Age, sex ratio, current smoking status, hypertension proportion, systolic blood pressure, STEMI proportion, and thrombolysis proportion were significantly different among the patients in the four groups. There were no differences in BMI, history of diabetes, or history of any other diseases (**Table 1**).

**Table 1:** Comparison of baseline data among the four groups [patients (%)].

Characteristic	TSH quartile μmol/L				P value
	Q1 (<1.02)	Q2 (1.02≤ ≤1.71)	Q3 (1.71-2.84)	Q4 (≥2.84)	
N=	878	886	880	873	
Age at randomization, year	61.4 (10.7)	60.5 (10.4)	61.4 (9.8)	62.7 (9.9)	0.0012
Female,n (%)	173 (19.7)	184 (20.8)	259 (29.4)	325 (37.2)	<0.0001
Past medical history					
Current smoker,n (%)	398 (45.3)	374 (42.2)	356 (40.5)	423 (48.5)	0.0041
Hypertension, n (%)	463 (52.7)	507 (57.2)	513 (58.3)	550 (63.0)	0.0003
Diabetes mellitus, n (%)	242 (27.6)	226 (25.5)	250 (28.4)	216 (24.7)	0.264
BMI, median (IQR), kg/m2	24.3 (3.3)	24.39 (3.0)	24.43 (3.2)	24.29 (3.0)	0.7803
Previous stroke, n (%)	61 (6.9)	51 (5.8)	39 (4.4)	58 (6.6)	0.1115
Renal insufficiency	36 (4.1)	31 (3.5)	34 (3.9)	42 (4.8)	0.5585
Heart failure	45 (5.1)	36 (4.1)	40 (4.5)	43 (4.9)	0.7287
History of atrial fibrillation, n (%)	32 (3.6)	28 (3.1)	34 (3.9)	34 (3.9)	0.8331
PCI (%)	102 (11.6)	96 (10.1)	119 (13.5)	100 (11.5)	0.3333
CABG (%)	4 (0.46)	4 (0.45)	3 (0.34)	1 (0.11)	0.5779
Clinical feature					
Systolic blood pressure, mm Hg	128.1 (21.0)	130.7 (20.4)	130.9 (19.0)	134.6 (21.2)	<0.0001
Diastolic blood pressure, mm Hg	77.4 (13.1)	77.2 (11.4)	76.54 (11.3)	77.7 (11.4)	0.2853
Killip classification					
I (%)	601 (68.4)	493 (55.6)	474 (53.8)	446 (51.1)	<0.0001
II (%)	171 (19.5)	223 (25.1)	256 (29.1)	226 (25.9)	<0.0001
III (%)	16 (1.8)	20 (2.3)	28 (3.2)	32 (3.7)	<0.01
IV (%)	7 (0.8)	7 (0.8)	2 (0.2)	9 (1.0)	0.2256
EF, % (SD)	52.98 (10.56)	51.93 (9.38)	52.91 (9.93)	52.35 (9.22)	0.6025
STEMI (% )	463 (52.7)	286 (32.3)	244 (27.7)	217 (24.9)	<0.0001
Thrombolysis (% )	133 (15.1)	82 (9.3)	85 (9.6)	80 (9.2)	<0.0001
Laboratory examination					
Fasting blood glucose, mmol/L (SD)	7.593 (3.4)	7.213 (3.4)	7.204 (3.5)	7.372 (3.8)	0.0691
Serum lipid					
Total cholesterol	3.916 (0.94)	3.804 (0.94)	3.815 (0.95)	3.958 (0.98)	0.3423
LDL cholesterol	2.313 (0.8)	2.216 (0.8)	2.221 (0.8)	2.332 (0.8)	0.6168
HDL cholesterol	0.9761 (0.2)	0.9656 (0.2)	0.9691 (0.2)	0.9909 (0.2)	0.185
Triglycerides	1.588 (1.1)	1.600 (1.0)	1.676 (1.1)	1.779 (1.3)	0.0002
HGB,g/L (SD)	138.6 (18.8)	139.9 (16.7)	137.8 (16.4)	135.4 (16.8)	<0.0001
WBC,10^9/L (SD)	8.547 (3.5)	7.156 (2.4)	6.964 (2.4)	6.849 (2.4)	<0.0001
CK-MB U/L (SD)	56.22 (98.4)	27.92 (53.4)	23.24 (42.7)	22.9 (51.9)	<0.0001
Scr umol/L (SD)	69.68 (31.0)	68.66 (42.3)	65.78 (26.0)	67 (20.2)	0.0195
Free thyroxine pmol/L, (SD)	4.406 (1.1)	4.606 (1.0)	4.6 (1.0)	4.608 (1.0)	0.0002
CRP,mg/L (SD)	4.99 (3.9)	3.31 (3.5)	3.17 (3.4)	3.17 (3.5)	<0.0001
hsTnT,ng/mL (SD)	0.8273 (1.72)	0.3443 (0.79)	0.3253 (0.83)	0.3444 (0.96)	<0.0001
Medication,n (%)					
Aspirin	792 (90.2)	820 (92.6)	806 (91.6)	793 (90.8)	0.3339
Plavix	615 (70.0)	637 (71.9)	621 (70.6)	611 (70.0)	0.8003
β-Blocker	650 (74.0)	666 (75.2)	651 (74.0)	654 (74.9)	0.9156
ACEI/ARB	643 (81.2)	710 (86.6)	699 (86.7)	689 (86.9)	0.0015
Statin	775 (88.3)	805 (90.9)	804 (91.4)	792 (90.1)	0.1235

Regarding laboratory tests, significant differences were found among the four groups in terms of triglyceride concentration, HGB, WBC, CK-MB, free thyroxine, CRP, and hs-cTnT. Except for the use of ACEIs, there was no significant difference in medication prescription among the four groups after hospitalization and discharge (**Table 1**).

### Clinical outcome

The median follow-up time was 70 (interquartile range=60-82) months, and the follow-up rate was 96.7%. Clinical adverse events occurred in 910 (25.9%) patients. Interestingly, we found that both elevated and low TSH levels within the normal range were associated with increased mortality and incidence of MACE.

The lowest mortality, incidence of MACE and heart failure were evident in Q2 patients. Besides, elevated TSH levels were also found to be associated with a higher incidence of revascularization events. The cardiac mortality rate was significantly lower in Q3 than in the other three groups. There was no significant difference in the incidence of myocardial infarction or stroke among the four groups (**Table 2**).

**Table 2:** Comparison of clinical adverse events in the four groups during the 5-year follow-up.

Comparison of clinical adverse events in the four group after 5 years follow-up, patients(%)						
Endpoint events	Total (n=3517)	Q1 (n=878)	Q2 (n=886)	Q3 (n=880)	Q4 (n=873)	P
MACE (%)	910 (25.9)	230 (26.2)	194 (21.9)	227 (24.7)	269 (30.8)	0.0004
All-cause mortailty	252 (7.2)	81 (9.2)	60 (6.8)	46 (5.2)	65 (7.5)	0.0124
cardiac death	209 (5.9)	72 (8.2)	43 (4.9)	41 (4.7)	53 (6.1)	0.0872
Myocardial infarction	138 (3.9)	37 (4.2)	34 (3.8)	30 (3.4)	37 (4.2)	0.7864
Revascularization	332 (9.4)	68 (7.7)	78 (8.8)	79 (9.0)	107 (9.0)	0.0236
Heart failure	262 (7.4)	60 (6.8)	38 (4.3)	79 (9.0)	95 (10.9)	<0.0001
Stroke	134 (3.8)	29 (4.3)	30 (3.4)	40 (4.5)	35 (5.2)	0.4855

**Figure 2:**
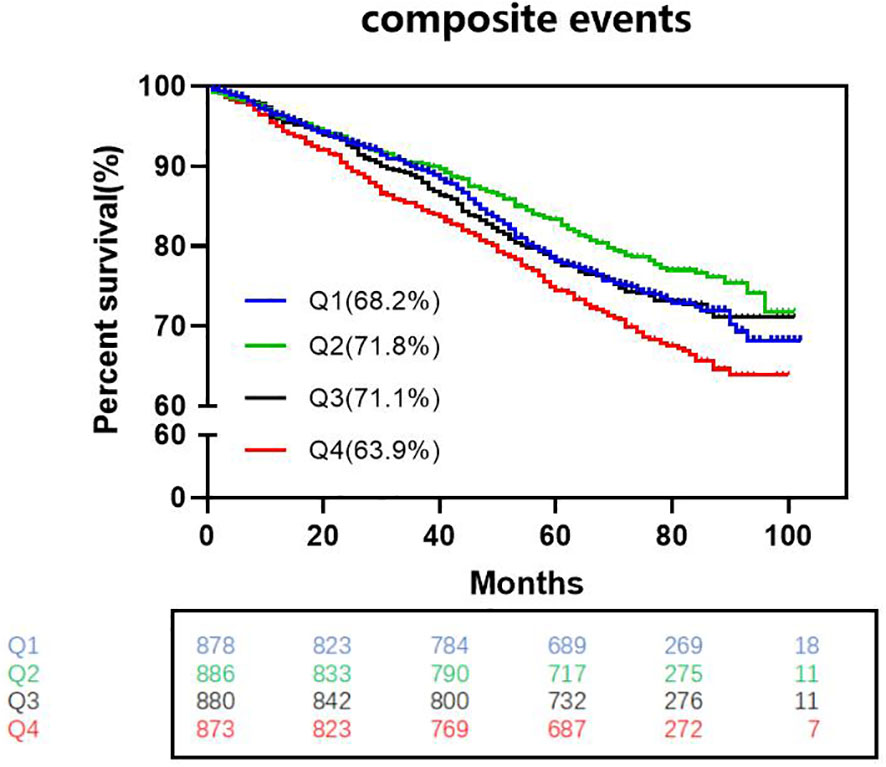
Kaplan-Meier survival curves without complex cardiovascular events among the four groups. (log-rank test, Q2 vs. Q1: Padj=0.0363, HR=0.815; Q2 vs. Q3: P.adj=0.034, HR=0.813; Q2 vs. Q4: P.adj<0.0001, HR=0.656) HR indicates hazard ratio (The p-values have already been adjusted using FDR).

K-M survival curve analysis revealed lower survival without composite adverse cardiovascular events in Q1, Q3, and Q4 patients than in Q2 patients. (log-rank test, Q2 *vs*. Q1: P.adj=0.0363, HR=0.815; Q2 *vs*. Q3: P.adj=0.034, HR=0.813; Q2 *vs*. Q4: P.adj<0.0001, HR=0.656) (**Figure 2**).

### The TSH levels have independent predictive value for MACE in patients

Cox regression analysis of risk ratios for adverse cardiovascular events among patients in the four groups (**Tables 3**, **4**): Cox multivariate regression models adjusted for other covariates (including age (<60, ≥60), male sex, hypertension, diabetes status, smoking status, and Killip class) showed that TSH in the upper part of the reference range was a significant predictor of the long-term occurrence of MACE. The highest TSH levels were associated with a greater risk of MACE than was the highest TSH levels (HR_Q4_ = 1.462, 95% CI=1.255-1.816, P=0.003). Moreover, patients with both a high TSH levels and a low TSH levels had an increased risk of heart failure compared with Q2 individuals (HR_Q1_ = 1.654, 95% CI=1.066-1.792, P=0.014; HR_Q3_ = 2.019, 95% CI=1.303-3.127, P=0.002; HRQ4 = 2.556, 95% CI=1.671-3.909, P=0.001).

**Table 3:** Univariate Cox regression analysis was used to analyze the hazard ratio (HR) of different TSH levels on adverse cardiovascular events.

Univariate Cox regression analysis was used to analyze the hazard ratio of different levels of TSH on adverse cardiovascular events							
Endpoint events	Q2 (n=886)	Q1 (n=878)		Q3 (n=886)		Q4 (n=873)	
		HR (95%CI)	P	HR (95%CI)	P	HR (95%CI)	P
MACE (%)	1	1.230 (1.016-2.164)	0.034	1.233 (1.018-1.494)	0.032	1.510 (1.255-1.816)	<0.0001
All-cause mortailty	1	1.379 (0.987-1.927)	0.059	0.772 (0.526-1.133)	0.186	1.109 (0.781-1.575)	0.564
cardiac death	1	1.20 (0.808-1.237)	0.361	0.649 (0.407-1.035)	0.069	1.001 (0.660-1.517)	0.998
Myocardial infarction	1	1.202 (0.732-1.972)	0.467	0.893 (0.546-1.459)	0.65	1.116 (0.701-1.778)	0.644
Revascularization	1	0.872 (0.630-1.208)	0.411	1.041 (0.762-1.422)	0.801	1.430 (1.068-1.915)	0.016
Heart failure	1	1.669 (1.112-2.506)	0.013	2.127 (1.445-3.133)	<0.001	2.678 (1.838-3.902)	<0.0001
Stroke	1	0.976 (0.586-1.626)	0.925	1.369 (0.853-2.197)	0.194	1.208 (0.742-1.967)	0.448

HR indicates hazard ratio (According to the formula for Bonferroni-corrected p-values, the original p-value must be less than 0.167 to achieve significance).

**Table 4:** Cox multivariate regression analysis was used to analyze the hazard ratios (HRs) of different TSH levels for adverse cardiovascular events (adjusted for age (<60, ≥60), male sex, hypertension, diabetes, smoking status, and Killip class (I and II, III and IV).

Univariate Cox regression analysis was used to analyze the hazard ratio of different levels of TSH on adverse cardiovascular events							
Endpoint events	Q2 (n=886)	Q1 (n=878)		Q3 (n=886)		Q4 (n=873)	
		HR (95%CI)	P	HR (95%CI)	P	HR (95%CI)	P
MACE (%)	1	1.198 (0.968-1.482)	0.097	1.230 (0.995-1.521)	0.056	1.462 (1.189-1.79)	0.003
All-cause mortailty	1	1.383 (0.956-2.002)	0.086	0.742 (0.481-1.145)	0.178	1.093 (0.739-1.617)	0.657
cardiac death	1	1.258 (0.811-1.961)	0.305	0.657 (0.309-1.105)	0.113	0.914 (0.569-1.468)	0.374
Myocardial infarction	1	1.081 (0.629-1.856)	0.779	0.989 (0.573-1.708)	0.97	1.253 (0.743-2.112)	0.398
Revascularization	1	0.756 (0.523-1.093)	0.137	1.100 (0.784-1.544)	0.581	1.390 (1.009-1.916)	0.044
Heart failure	1	1.654 (1.046-2.616)	0.031	2.019 (1.303-3.127)	0.002	2.556 (1.671-3.909)	0.001
Stroke	1	0.993 (0.578-1.705)	0.979	1.234 (0.739-2.058)	0.421	1.169 (0.683-2.00)	0.569

HR indicates hazard ratio. (According to the formula for Bonferroni-corrected p-values, the original p-value must be less than 0.167 to achieve significance).

### There are sex differences in TSH levels for predicting heart failure

**Figure 3:**
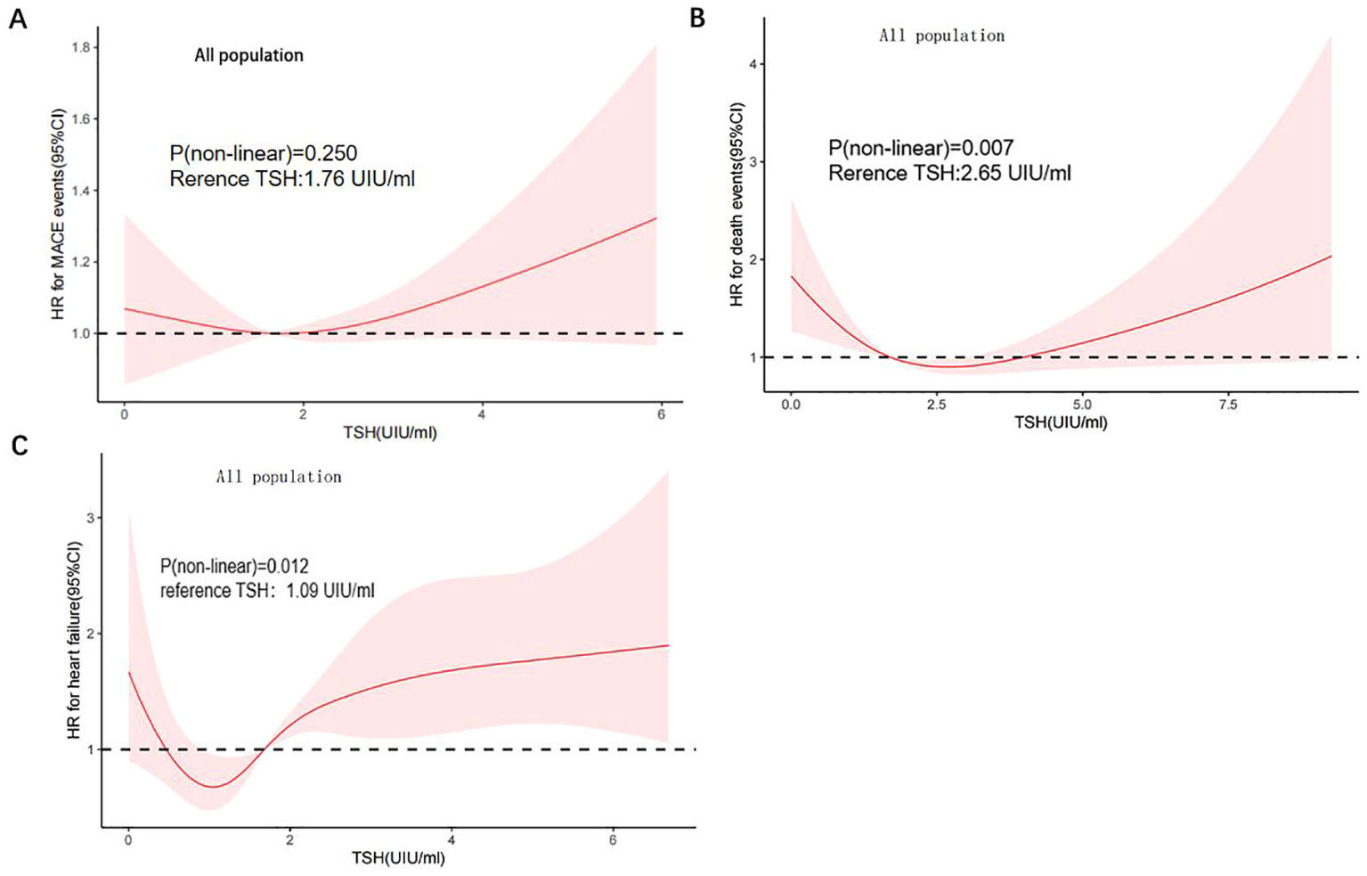
A restricted cubic spline regression model. **(A)** TSH with all-cause mortality; **(B)** TSH with MACE; **(C)** TSH with heart failure) The results were adjusted for age, smoking status, cancer history, and estimated glomerular filtration rate. A restricted cubic spline regression model was constructed with 4 nodes at the 5th, 35th, 65th and 95th percentiles of TSH. The dotted lines represent the 95% confidence intervals for the spline model. The range of TSH should be restricted to 0.34 to 6.5 mIU/L because predictions greater than 6.5 mIU/L (95th percentile) are based on too few data points. HR indicates hazard ratio.

**Figure 4:**
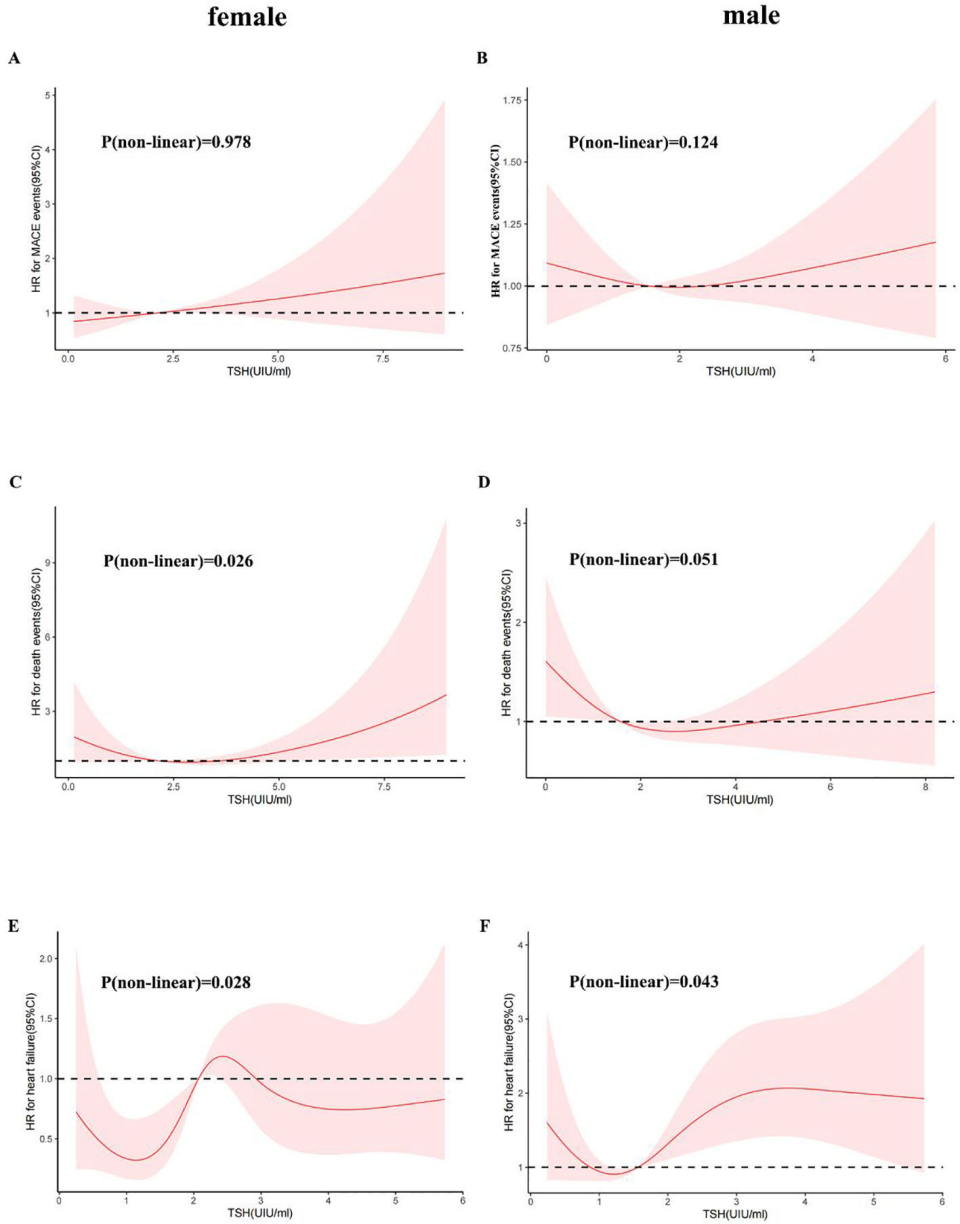
A restricted cubic spline regression model was used for sex. (Female: **(A)** TSH with all-cause mortality; **(C)** TSH with MACE events; **(E)** TSH with heart failure; Male: **(B)** TSH with all-cause mortality; **(D)** TSH with MACE events; **(F)** TSH with heart failure) The results were adjusted for age, smoking status, cancer history, and estimated glomerular filtration rate. A restricted cubic spline regression model was constructed with 4 nodes at the 5th, 35th, 65th and 95th percentiles of TSH. The dotted lines represent the 95% confidence intervals for the spline model. The range of TSH should be restricted to 0.34 to 6.5 mIU/L because predictions greater than 6.5 mIU/L (95th percentile) are based on too few data points. HR indicates hazard ratio.

We further utilized a restricted quartic spline to build a flexible model, visually illustrating the relationship between TSH concentrations within the reference range and MACE in patients with CHD (**Figure 3A**). Elevated TSH levels, regardless of gender, were strongly associated with an increased risk of MACE (**Figures 4A, B**).

A higher serum TSH concentration above the median was linked to an increase in all-cause mortality, and mortality continued to rise with increasing TSH levels (**Figure 3B**). Low TSH concentrations were also associated with higher mortality, though the differences between sexes were minimal (**Figures 4C, D**).

TSH levels in both the upper and lower regions of the reference range were connected to an increased risk of heart failure (**Figure 3C**). In females, lower TSH concentrations were negatively associated with the risk of heart failure, while in males, both high and low TSH concentrations were positively associated with heart failure risk (**Figures 4E, F**).

## Discussion

The present study assessed the impact of fasting serum TSH levels at admission in patients with coronary heart disease on long-term MACE and all-cause mortality (for a median follow-up of 70 months). The most important findings of the study can be summarized as follows: 1). There was an increased risk of all-cause mortality and MACE among patients in the higher TSH levels group compared to patients in the other groups. 2). TSH in the upper and lower regions of the reference range is an independent predictor of increased risk of heart failure. 3). TSH is a risk factor for MACE, and there are sex differences in HF.

This study revealed that a higher TSH levels were independent predictor of MACE for patients with CHD, which is consistent with the findings of several previous studies (21). Previous studies have also reported that subclinical hypothyroidism is associated with an increased risk of CHD events, CHD mortality and CHD severity in individuals with higher TSH levels (20, 26–28).Even in patients with CHD who underwent PCI, TSH levels in the upper part of the reference range was also associated with an increased risk of mortality after PCI (20). Some studies suggest that subclinical hyperthyroidism, as identified by a reduced TSH level, is an important risk factor for cardiac events, mortality, and the development of atrial fibrillation (29). However, our study revealed that there was no independent association between lower TSH levels and cardiac events or mortality. The incidence of STEMI and thrombolysis was greater in the baseline data of the lower TSH group than in those of the other groups, which may have affected the clinical prognosis of the patients. Thus, the correlation between lower TSH levels and MACE disappeared after correction for multivariate Cox analysis.

This study also revealed that the TSH levels within the upper and lower limits of the reference range is an independent predictor of an increased risk of heart failure. A population-based survey of 4,987 patients revealed that the elevated TSH levels were associated with heart failure (30). Individual participant data analyses of prospective cohort studies with 25390 participants from the International Thyroid Studies Collaboration showed that heart failure risk increased with both higher and lower TSH levels (13).This finding is consistent with our findings.

This study revealed that the TSH concentration is a risk factor for MACE. We used a restricted quartic spline to visualize the association between TSH concentration and MACE and all-cause death in CHD patients. For females, TSH concentrations were linearly positively associated with the risk of MACE. For males, with increasing TSH concentration, the hazard ratio (HR) first increased and then decreased (**Figures 4A, B**). TSH concentrations were within the reference range for heart failure and for CHD patients. For all populations above 1.09 mIU/L, the TSH concentration was positively associated with the risk of heart failure (**Figure 3C**). Although the HRs for men and women were similar to that for TSH, men with the same TSH levels had a significantly greater risk of heart failure (**Figures 4E, F**). Therefore, the TSH levels within the normal range are more predictive of long-term heart failure events in male CHD patients.

There are a few reasons for the prognostic influence of TSH levels in patients with CHD in this study. Previously, studies have demonstrated that the correlation between TSH and the cardiovascular system includes changes in arterial compliance, diastolic blood pressure, endothelial dysfunction, vascular resistance, and cardiac contractility (22–24, 31). Other cardiovascular effects include myocardial damage; pericardial effusion; and metabolic syndrome-related factors, including hypertension, increased dyslipidemia, and waist circumference (32, 33). The TSH levels were found to be independently correlated with both carotid plaque incidence and intima-media thickness, and TSH can contribute to atherogenesis directly by promoting macrophage inflammation in atherosclerotic (31, 34). In addition, TSH was found to be positively related to serum lipid concentration (35). High serum levels of TSH accelerate the production of inflammatory molecular and cardiovascular risk biomarkers, increasing the risk of cardiovascular diseases (36). An increase in inflammatory reactions accelerates atherosclerosis and heart failure. It is obvious that TSH directly affects many cardiovascular system physiological processes. However, further studies are needed to elucidate the mechanism by which different TSH levels independently predict adverse outcomes in the CHD population.

Therefore, TSH is not only a risk marker but also a risk target that should be considered to decrease adverse cardiovascular outcomes.

The strength of our study is that we continuously enrolled patients according to the seven-year CAG results. This method significantly decreases the potential misdiagnosis. In addition, this was a single-center study. Consistent detection methods for TSH and other clinical indicators can reduce errors caused by differences in methods and detection standards. This study has several limitations. First, this was a single-center study restricted to Chinese Shaanxi patients. Therefore, generalizing our findings to other ethnic groups requires further research on different ethnic groups to support our findings. Second, this study only examined a single TSH level during hospitalization, lacking dynamic TSH data. Third, patient information extracted from medical records was used, and its completeness and accuracy depended on the physician. Furthermore, this was an observational study. Therefore, the possibility of error cannot be ruled out. However, further prospective studies are needed to support our findings.

## Conclusion

In patients with CHD, TSH levels in the upper and lower parts of the reference range are associated with an increased risk of long-term MACE and heart failure. TSH levels have independent predictive value for adverse cardiovascular events and heart failure in patients, especially male patients, with CHD. Additionally, screening TSH levels may help improve risk classification and treatment outcomes for CHD patients. Future studies are needed to clarify this relationship and explore whether treatments targeting TSH improvement can reduce the occurrence of adverse events.

## Data Availability

The raw data has been stripped of sensitive information and added to the supplementary materials, with full public access.
